# Polyyne production is regulated by the transcriptional regulators PgnC and GacA in *Pseudomonas protegens* Pf-5

**DOI:** 10.1128/aem.02388-24

**Published:** 2025-04-03

**Authors:** Chiseche Mwanza, Maria Purnamasari, Daniel Back, Cahya Prihatna, Benjamin Philmus, Khaled H. Almabruk, Taifo Mahmud, Lumeng Ye, Melvin D. Bolton, Xiaogang Wu, Joyce E. Loper, Qing Yan

**Affiliations:** 1 Department of Plant Sciences and Plant Pathology, Montana State University 169020 , Bozeman, Montana, USA; 2 Edward T. Schafer Agricultural Research Center, US Department of Agriculture, Agricultural Research Service 17123, Fargo, North Dakota, USA; 3 Department of Pharmaceutical Sciences, Oregon State University 540632, Corvallis, Oregon, USA; 4 Institute of Molecular Biology and Biotechnology, Vrije Universiteit Brussel 129259 , Brussels, Belgium; 5 College of Agriculture, Guangxi University 12664, Nanning, China; 6 Horticultural Crops Research Laboratory, US Department of Agriculture, Agricultural Research Service 17123, Corvallis, Oregon, USA; University of Tennessee at Knoxville, Knoxville, Tennessee, USA

**Keywords:** polyyne biosynthesis, *Pseudomonas protegens*, gene regulation, antimicrobial activity

## Abstract

Polyynes produced by bacteria have promising applications in agriculture and medicine due to their potent antimicrobial activities. Polyyne biosynthetic genes have been identified in *Pseudomonas* and *Burkholderia*. However, the molecular mechanisms underlying the regulation of polyyne biosynthesis remain largely unknown. In this study, we used a soil bacterium *Pseudomonas protegens* Pf-5, which was recently reported to produce polyyne called protegenin, as a model to investigate the regulation of bacterial polyyne production. Our results show that Pf-5 controls polyyne production at both the pathway-specific level and a higher global level. Mutation of *pgnC*, a transcriptional regulatory gene located in the polyyne biosynthetic gene cluster, abolished polyyne production. Gene expression analysis revealed that PgnC directly activates the promoter of polyyne biosynthetic genes. The production of polyyne also requires a global regulator GacA. Mutation of *gacA* decreased the translation of PgnC, which is consistent with the result that *pgnC* leader mRNA bound directly to RsmE, an RNA-binding protein negatively regulated by GacA. These results suggest that GacA induces the expression of the PgnC regulator, which in turn activates polyyne biosynthesis. Additionally, the polyyne-producing strain of Pf-5, but not the polyyne-nonproducing strain, could inhibit a broad spectrum of bacteria including both Gram-negative and Gram-positive bacteria.

**IMPORTANCE:**

Antimicrobial metabolites produced by bacteria are widely used in agriculture and medicine to control plant, animal, and human pathogens. Although bacteria-derived polyynes have been identified as potent antimicrobials for decades, the molecular mechanisms by which bacteria regulate polyyne biosynthesis remain understudied. In this study, we found that polyyne biosynthesis is directly activated by a pathway-specific regulator PgnC, which is induced by a global regulator GacA through the RNA-binding protein RsmE in *Pseudomonas protegens*. To our knowledge, this work is the first comprehensive study of the regulatory mechanisms of bacterial polyyne biosynthesis at both pathway-specific level and global level. The discovered molecular mechanisms can help us optimize polyyne production for agricultural or medical applications.

## INTRODUCTION

Bacteria-derived secondary metabolites have broad applications in agriculture and human medicine (1, 2). Production of bacterial secondary metabolites can vary greatly under different growth conditions and is regulated by various genetic and environmental factors (3). Understanding the molecular mechanisms that regulate secondary metabolite production can help us better understand their natural roles and optimize their production for agricultural or medical applications (4).

Polyynes are secondary metabolites characterized by the presence of multiple carbon-carbon triple bonds (also called alkyne bonds). Over the past 50 years, hundreds of polyyne derivatives were identified from bacteria, fungi, insects, and plants (5, 6). Polyynes have potent antimicrobial activities with promising applications in the treatment of bacterial and fungal pathogens of plants, animals, and humans. For example, polyyne derivatives produced by *Burkholderia* spp. have a broad antimicrobial spectrum including Gram-positive bacteria, fungi, and oomycetes (7–9). Additionally, polyynes isolated from some plants, such as *Bidens pilosa*, have antidiabetic, antiviral, and anti-inflammatory activity (10). However, research and applications of polyynes are limited by their unstable nature and the low yields of their natural producers (10). Improving polyyne production through genetic engineering is of interest and requires an advanced understanding of the molecular mechanisms that regulate its biosynthesis.

Bacteria-derived polyynes were first discovered from the human pathogen *Burkholderia diffusa* (previously known as *Pseudomonas cepacia* or *Burkholderia cepacia*) (11). Since then, diverse polyyne derivatives have been identified from different bacteria including the plant pathogen *Trinickia caryophylli* (previously known as *Pseudomonas caryophylli* or *Burkholderia caryophylli*) (12), actinomycete *Microbispora* sp. (13), the cyanobacterium *Fischerella muscicola* (14), the fungus-feeding bacterium *Collimonas fungivorans* (15), the marine bacterium *Gynuella sunshinyii* (16), the biopesticidal bacterium *Burkholderia ambifaria* (7), and the plant beneficial bacteria *Pseudomonas protegens* (17–20).

The bacterial polyyne biosynthetic gene cluster was first identified in *T. caryophylli* (21) and later was characterized in other polyyne-producing bacteria including *B. ambifaria*, *Massilia* sp., and *P. protegens* (7, 8, 18). The core biosynthetic enzymes for bacterial polyyne production include a fatty acyl-AMP ligase, a fatty acid desaturase, and an acyl carrier protein (22). Polyyne biosynthesis is initiated by the ligase-catalyzed activation and loading of a fatty acid onto an acyl carrier protein, followed by desaturation and then releasing the polyyne product from the acyl carrier protein (23).

Compared to the relatively well-studied polyyne biosynthesis, much less is known about the molecular mechanisms that regulate bacterial polyyne production. Potential regulatory genes were annotated in polyyne biosynthetic gene clusters, but their roles in the regulation of polyyne production remain largely unknown. The polyyne biosynthetic gene cluster of *B. ambifaria* contains *luxI/luxR* family regulatory genes, but their roles in polyyne production were not characterized (7). The polyyne biosynthetic gene cluster of *C. fungivorans* has a *lysR*-type regulatory gene. Mutation of the *lysR* gene reduced the antifungal activity of *C. fungivorans* (15), although the underlying mechanism was not investigated. A *P. protegens* Cab57 mutant lacking *gacS*, which encodes a sensor kinase of the GacS/GacA global regulatory system, lacks polyyne production (20). The GacS/GacA system regulates the expression of target genes mainly at posttranscriptional levels through a signaling pathway that includes small regulatory RNAs and RNA-binding proteins (24). For example, GacA induces the expression of three regulatory RNAs, *rsmX*, *rsmY*, and *rsmZ*, which sequester the RNA-binding proteins RsmA and RsmE and thereby relieve translational repression exerted by these proteins in *P. protegens* CHA0 (25) and *P. protegens* Pf-5 (26). The mechanism by which GacS/GacA regulates polyyne production has not been elucidated.

**Fig 1:**
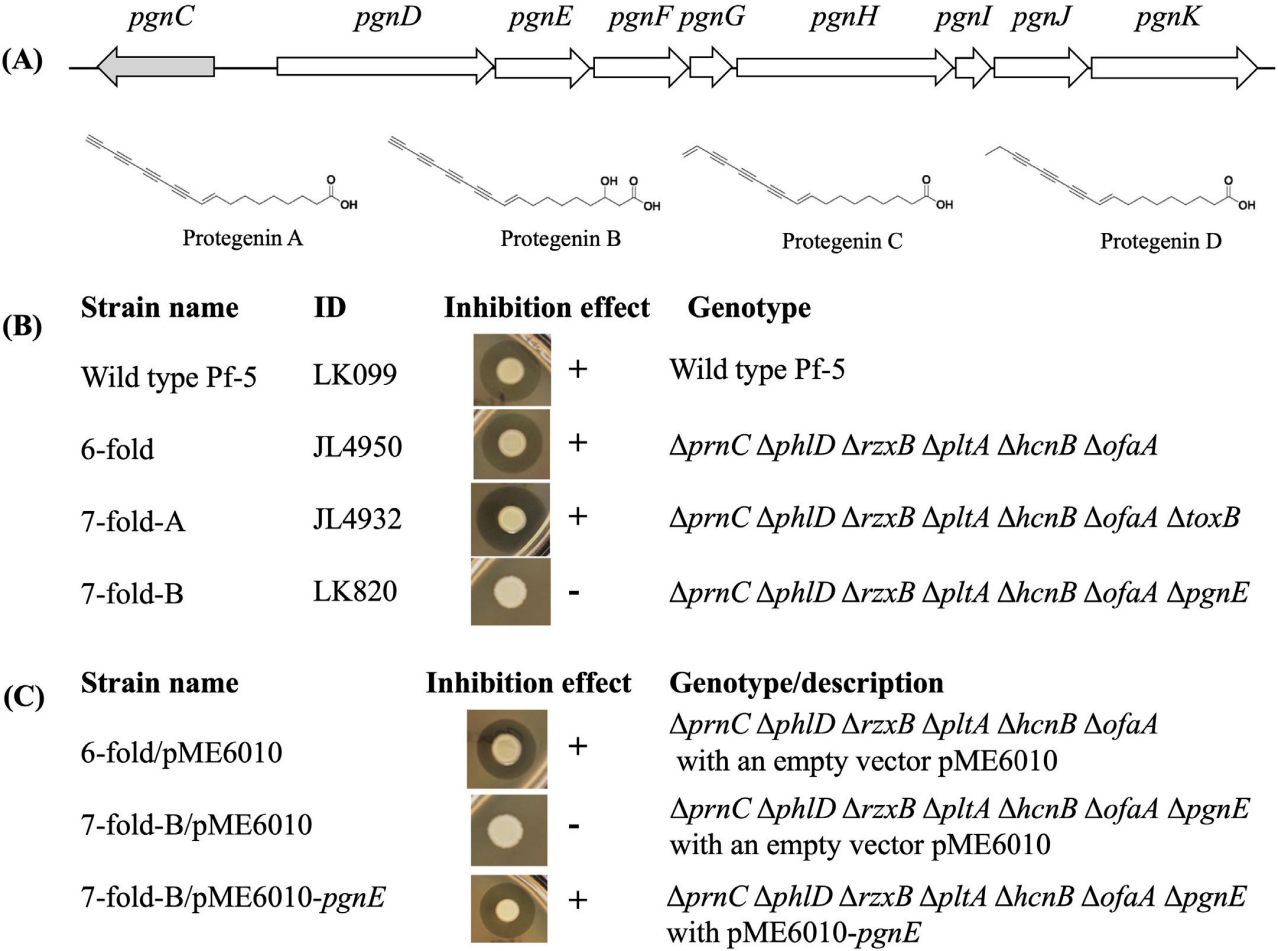
*P. protegens* Pf-5 produces polyyne that inhibits the growth of *Pseudomonas syringae* DC3000 on NAGly plates. (A) Polyyne biosynthetic gene cluster of Pf-5. The regulatory gene *pgnC* is shown in gray color. Chemical structures of polyyne derivatives Protegenin A–D were shown. Protegenin A, C, and D were detected from Pf-5 cultures in this study and a previous report (19). (B) Inhibition of wild-type Pf-5, the sixfold, sevenfold-A, and sevenfold-B mutants against *P. syringae* DC3000 on NAGly. (C) Inhibition of the sixfold mutant, the sevenfold-B mutant, and its complementation strain against *P. syringae* DC3000 on NAGly. “+” and “−” Indicate inhibition and no inhibition, respectively. The experiments were repeated at least three times, which generated similar results.

To advance our understanding of the molecular mechanisms that regulate polyyne production, we used the soil bacterium *P. protegens* Pf-5, which serves as a model strain to study bacterial secondary metabolites due to its production of many antimicrobials including pyrrolnitrin, hydrogen cyanide, 2,4-diacetyalphloroglucinol, pyoluteorin, rhizoxin, orfamide A, and toxoflavin (27–30). Recently, several polyyne derivatives, called protegenins (also called protegencins), were identified from Pf-5 (Fig. 1) (17–19). The polyyne gene cluster of Pf-5 encodes a putative pathway-specific regulator PgnC. In this study, the role of PgnC in polyyne biosynthesis was characterized. Furthermore, the mechanism by which the global regulator GacS/GacA controls polyyne production was investigated, and the connection between the global regulation and pathway-specific regulation was discussed.

This study also investigated antibacterial activity of the polyynes (protegenins) produced by Pf-5. Protegenin A was previously found to be toxic to the alga *Chlamydomonas reinhardtii* (17). Protegenin A and B, isolated from *P. protegens* Cab57, were shown to have antifungal and anti-oomycete activities (20). However, the antibacterial activities of the protegenins have not been characterized. To fill this knowledge gap, different bacteria including Gram-positive and Gram-negative strains were tested in the antagonistic assays using polyyne-producing strain and nonproducing strain of Pf-5.

## MATERIALS AND METHODS

### Strains and cultural conditions

The bacterial strains, plasmids, and sequences of oligonucleotides used in this study are listed in Table 1. *P. protegens* Pf-5 and its mutants were cultured at 28°C on King’s Medium B (KB) agar (31), nutrient agar (Becton, Dickinson and Company, Sparks, MD) supplemented with 1% glycerol (NAGly), or nutrient broth (Becton, Dickenson, and Company) supplemented with 1% glycerol (NBGly). Liquid cultures were grown with shaking at 200 rpm (revolutions per minute). Antibiotics were added at different concentrations as needed (Table 1).

**TABLE 1:** Bacterial strains, plasmid, and primers used in this study

Strain, plasmid, or primer	Genotype, relevant characteristics, or DNA sequence (5′ to 3′)^*a*^	Reference or source
Strains		
*Agrobacterium tumefaciens*	Strain C58, a bacterial pathogen causing crown gall disease in plants	(32)
*Bacillus subtilis*	Strain 168, a gram-positive bacterium widely used as a model organism for basic research in molecular biology	(33)
*Chryseobacterium* sp.	Bacterial isolate from pea rhizosphere soils	(30)
*Erwinia amylovora*	Strain MEa141, isolated from apples in Montana, causes apple fire blight disease	Lab collection
*Escherichia coli*		
SW105	Genetically modified *E. coli* widely used in molecular biology research	Lab collection
BL21(DE3)	Genetically modified *E. coli* commonly used for protein overexpression, *ompT hsdS*_B_ (rB^-^ mB^-^) *gal dcm*	NEB
*Paraburkholderia phytofirmans*	Strain PsJN, isolated from onion root, is a plant symbiotic bacterium that promotes the growth and fitness of several plant species, including pea	(34, 35)
*Pseudomonas* spp.		
*Pseudomonas fluorescens*		
SBW25	Member of the *P. fluorescens* group, does not contain the *pgn* gene cluster for polyyne biosynthesis	(36)
2P24	Isolated from wheat take-all natural decline soils, produces DAPG and has biocontrol effect on bacterial wilt disease on tomato	(37)
*P. protegens*		
Wild-type Pf-5	Soil bacterium; assigned ID is LK099; wild type; Prn^+^, DAPG^+^, Rzx^+^, Plt^+^, HCN^+^, Ofa^+^, Tox^+^, Pgn^+^	(38)
∆*gacA* mutant	Pf-5 derivative; assigned ID is JL4975; ∆*gacA*, altered in the many phenotypes regulated by GacA, Prn^−^, DAPG^−^, Rzx^−^, Plt^−^, HCN^−^, Ofa^−^, Tox^−^, Pgn^−^	(39)
Sevenfold-A mutant	Pf-5 derivative; assigned ID is JL4932; ∆*prnC* ∆*phlD* ∆*rzxB* ∆*pltA* ∆*hcnB* ∆*ofaA* Δ*toxB*; Prn^−^, DAPG^−^, Rzx^−^, Plt^−^, HCN^−^, Ofa^−^, Tox^−^	(40)
Sixfold mutant	Pf-5 derivative; assigned ID is JL4950; ∆*prnC* ∆*phlD* ∆*rzxB* ∆*pltA* ∆*hcnB* ∆*ofaA*; Prn^−^, DAPG^−^, Rzx^−^, Plt^−^, HCN^−^, Ofa^−^	This study
Sevenfold-B mutant	Pf-5 derivative; assigned ID is LK820; ∆*prnC* ∆*phlD* ∆*rzxB* ∆*pltA* ∆*hcnB* ∆*ofaA* Δ*pgnE*; Prn^−^, DAPG^−^, Rzx^−^, Plt^−^, HCN^−^, Ofa^−^, Pgn^−^	This study
Sevenfold-C mutant	Pf-5 derivative; assigned ID is LK620; ∆*prnC* ∆*phlD* ∆*rzxB* ∆*pltA* ∆*hcnB* ∆*ofaA* Δ*pgnC*; Prn^−^, DAPG^−^, Rzx^−^, Plt^−^, HCN^−^, Ofa^−^, Pgn^−^	This study
*Pseudomonas putida*	Strain KT2440, a soil bacterium, has been certified as a biosafety host for the expression of foreign genes	(41)
*Pseudomonas syringae*		
*P. s*. pv. *syringae*	Strain B728, a pathogenic bacterium, causes leaf diseases on many plants including tomato and Arabidopsis	(42)
*P. s*. pv. *tomato*	Strain DC3000, a pathogenic bacterium, causes leaf diseases on many plants including tomato and Arabidopsis	(42)
*Staphylococcus aureus*	Strain ATCC 12600, an opportunistic pathogen and frequent colonizer of the epithelium causing diseases in humans and animals	American Type Culture Collection
*Xanthomonas translucens*	Strain MW40, isolated from barley in Montana, causes bacterial leaf streak on barley	Lab collection
Plasmids		
pEX18Tc	Suicide vector in *P. protegens*; contains MCS from pUC18; used to make deletion and gene replacement constructs of Pf-5; *sacB*^+^ Tc^r^	(43)
pME6010	Expression vector in *P. protegens*; Tc^r^	(44)
pEX18Tc-Δ*pgnE*	pEX18Tc containing an in-frame deleted version of the *pgnE* gene; used to delete *pgnE* in the chromosome of Pf-5; assigned ID is LK818	This study
pEX18Tc-∆*pgnC*	pEX18Tc containing an in-frame deleted version of the *pgnC* gene; used to delete *pgnC* in the chromosome of Pf-5; assigned ID is LK615	This study
pME6010-*pgnE*	pME6010 containing the wild-type *pgnE* gene; used to restore *pgnE* in the Δ*pgnE* mutant; assigned ID is LK821	This study
pME6010-*pgnC*	pME6010 containing the wild-type *pgnC* gene; used to restore *pgnC* in the Δ*pgnC* mutant; assigned ID is LK757	This study
pPROBE-NT	Empty vector used to make reporter constructs; contains a promoterless *gfp*, Km^r^	(45)
p*pgnD*_promoter_:*gfp*	pPROBE-NT containing the *pgnD* promoter fused in-frame with a promoterless *gfp* gene; used to measure the transcriptional expression of the *pgnD* gene; assigned ID is LK641	This study
p*pgnC*_promoter_:*gfp*	pPROBE-NT containing the *pgnC* promoter fused in-frame with a promoterless *gfp* gene; used to measure the transcriptional expression of the *pgnC* gene; assigned ID is LK642	This study
p*pgnC-pgnD*_promoter_:*gfp*	pPROBE-NT containing the wild-type gene *pgnC* and the promoter of *pgnD* fused with a promoterless *gfp* gene; used to measure the transcriptional expression of *pgnD* with the presence of PgnC regulator; assigned ID is LK756	This study
pPgnC_translation_:*rfp*	pPROBE-NT containing the *pgnC* promoter, the 5’ untranslated region, and the first 10 codons of *pgnC* fused in-frame with a promoterless *rfp* gene cloned from pBbE1a; used to measure the translational expression of the *pgnC* gene; assigned ID is LK797	This study
Primers		
PFL_0262f1	ATAGGTACCTGATCATCATTGCCGGG	
PFL_0262r1	TATAAGCTTGAACGCACGATGGGCCAG	
PFL0260_F2	ATAGAATTCATTCAAAATCCTTTTTAAATG	
PFL0260_R2	TATAAGCTTCCAGCGCAATAAGCGTGAG	
PFL0260_R3	ATATCTAGATGATCGGCGGCCCGG	
PFL0260_R4	ATAGAATTCCAGCGCAATAAGCGTG	
PFL0260_R5	ATAGGTACCTAGATGATCGGCGGCCC	
RFP_0260_OVLP	CTTCGCTACTCGCCATTTCCAGCGCAATAAGCGTGAG	
GFP_2061_OVLP	GTTCTTCTCCTTTACTCATAAGATAATCACGAGGCACGGC	
RFP_F1	TCGACTCTAGAGTTAAGCACCGGTGGAGTGAC	
RFP_R1	CTTATTGCGCTGGAAATGGCGAGTAGCGAAGACG	
P35_RFP	CACTCCACCGGTGCTTAACTCTAGAGTCGACCTGCAGG	
P34_0261	TCGTGATTATCTTATGAGTAAAGGAGAAGAACTTTTCACTG	
PFL_0260 UpHindIII	TATAAGCTTCCACAGTTCGGCGTAGC	
PFL_0260 UpR	GCAGCCGGGGCCTAGATGATCAGCGCAATAAGCGTGAGC	
PFL_0260 DnF	GCTCACGCTTATTGCGCTGATCATCTAGGCCCCGGCTGC	
PFL_0260 DnEcoRI	ATAGAATTCGGCGCCTGATCATCTCGG	
PFL2095_F1	GAAGGAGATATACCATGCTGATACTCACCCGCAAAG	
PFL2095_R1	TGGTGGTGGTGGTGGTGGGGGGTTTCGCGTTTGTCCG	
28aPFL2095_F1	GGTGAGTATCAGCATGGTATATCTCCTTCTTAAAG	
28aPFL2095_R1	CGCGAAACCCCCCACCACCACCACCACCACTGAG	
pgnC_UTR_F	TAATACGACTCACTATAGGGCACAGGGAATAGCGTGGGCAG	
pgnC_UTR_R	CGATCCCATGGCACCTTCCAG	

^*a*^ Phenotype abbreviations: DAPG, 2,4-diacetylphloroglucinol; HCN, hydrogen cyanide; Ofa, orfamide A; Plt, pyoluteorin; Prn, pyrrolnitrin; Rzx, rhizoxin derivatives; Pgn, polyyne protegenins; Tox, toxaflavins. Abbreviations of antibiotics and their concentrations used in this work: Tc, tetracycline (10 µg/mL for *E. coli*, 200 µg/mL for Pf-5); Km, kanamycin (50 µg/mL). For DNA sequences, underlining shows DNA restriction enzyme sites that were used for cloning.

### Construction of Pf-5 mutants and complementation strains

Pf-5-derived deletion mutants were made by following our previous method (46). Briefly, to delete the *pgnC*, two DNA fragments flanking the *pgnC* gene were PCR amplified using the oligonucleotide pair PFL_0260 UpHindIII/PFL_0260 UpR and PFL_0260DnF/PFL_0260 DnEcoRI (Table 1). These two fragments were fused together by PCR and digested using *Eco*RI plus *Hin*dIII to generate a 1,138 bp DNA fragment containing *pgnC* with a 1,010 bp in-frame deletion. This DNA fragment was ligated to a suicide vector pEX18Tc, which carries a *tetR* gene for tetracycline resistance and a *sacB* gene for sucrose selection, to create the deletion construct pEX18Tc-Δ*pgnC*. The deletion construct was confirmed by Sanger-sequencing analysis and introduced into Pf-5 derivatives via electroporation (1.8 kV, 5 ms). The transformants were cultured on King’s Medium B agar amended with tetracycline to select recombinants followed by counterselection on agar plates amended with sucrose but without tetracycline. Colonies formed on the sucrose-amended plates were screened by PCR analysis to identify mutants that have a deletion of the *pgnC* gene.

The *pgnE* mutant was made by deleting *pgnE* from the chromosome of Pf-5 derivatives. To make the *pgnE* deletion construct, a 1,616 bp DNA fragment, which harbors the *pgnE* gene and its flanking regions, was amplified from the wild type of Pf-5’s genome using primers PFL_0262f1 paired with PFL_0262r1. The PCR product was digested by *Kpn*I and *Hin*dIII and ligated into pEX18Tc. A 356 bp fragment of the *pgnE* internal fragment was removed from the construct using *Sph*I digestion. The deletion construct was confirmed by Sanger-sequencing analysis and then introduced into Pf-5 derivatives via electroporation to make mutants that have a deletion of the *pgnE* gene as described above.

To restore the deleted gene, a complementation construct was made using the expression vector pME6010 and transformed into the Pf-5 mutants. Specifically, to restore *pgnC*, a 1,334 bp DNA fragment containing the wild-type *pgnC* gene was amplified using primers PFL0260_F2 and PFL0260_R5, digested with *Eco*RI and *Kpn*I, and ligated into a similarly digested pME6010 to make the complementation construct pME6010-*pgnC*. Similarly, to restore *pgnE*, a 1,616 bp DNA fragment containing the wild-type *pgnE* gene was amplified using primers PFL_0262f1 and PFL_0262r1, digested with *Kpn*I plus *Hin*dIII, and ligated into a similarly digested pME6010 to make the complementation construct pME6010-*pgnE*. The generated constructs were confirmed by Sanger sequencing and transformed into Pf-5 mutants via electroporation (1.8 kV, 5 ms) to restore the genes.

### Antibacterial activity assays

Pf-5 strains, including both polyyne-producing and nonproducing derivatives, and the target-tested bacterial strains (for example *Pseudomonas syringae*) were cultured overnight on NAGly plates at 28°C. The bacterial cells were collected from the culture plates and washed into 1 mL water solution that was then diluted to a final optical density at 600 nm (OD_600_) of 1.0. The target-tested bacterial strains were inoculated into melted warm (around 45°C) NBGly medium at a final OD_600_ of 0.01. The medium-cell mixture was then plated on a petri dish and solidified by air-drying for 10–15 minutes in a biosafety cabinet.

To test the antagonistic activity of the polyyne-producing and nonproducing Pf-5 derivatives, 3 µL of the Pf-5 bacterial suspension (OD_600_ = 1.0) was inoculated on the solidified NAGly plates that were pre-mixed with the target-tested bacterial cells described above. The plates were air-dried in the laminar flow hood and incubated at 28°C for around 24 hours before the inhibition zones of Pf-5 to the tested bacterial strains were measured and recorded.

The experiment had three replications for each treatment and was repeated at least three times independently.

### High-performance liquid chromatography and liquid chromatography mass spectrometry analyses

*P. protegens* Pf-5 strains were cultured in 2 mL of NBGly at 28°C for 16 h (200 rpm). The bacterial cells were collected from the cultures and adjusted to OD_600_ = 1.0 with fresh NBGly. A portion (100 µL) of the adjusted inoculum was spread onto NAGly plates and incubated at 28°C. After 24 h, the plates were cut into pieces and extracted with ethyl acetate (EtOAc; 200 mL) for 2 h (shaking at 100 rpm). The EtOAc extracts were then decanted, 0.5 mL of dimethyl sulfoxide (DMSO) was added, and the EtOAc was removed under reduced pressure. The resulting DMSO solution was used directly for high-performance liquid chromatography (HPLC) analysis.

HPLC data were obtained using a Thermo Scientific Dionex Ultimate 3000 HPLC system (Waltham, MA), consisting of a vacuum degasser, quaternary pump, thermostatted column compartment (maintained at 22°C), and an autosampler (maintained at 10°C) coupled to a Waters 996 photodiode array detector (Milford, MA). Chromatographic separation was achieved using a Luna C18 (2) column (250 × 10 mm, 5 µm, Phenomenex, Torrance, CA) at a flow rate of 2.5 mL/min. The solvents used were water (solvent A) and acetonitrile (solvent B). The column was equilibrated with 90% A/10% B. Upon injection, the mobile phase composition was maintained for 1 minute followed by changing the mobile phase to 0% A/100% B over 19 minutes using a linear gradient. The mobile phase was held at 0% A/100% B for 15 minutes. The mobile phase was then changed back to 90% A/10% B, and the column equilibrated for 7 minutes prior to the next injection. HPLC operation and data observation were conducted using Chromeleon (version 7.2, Thermo Fisher Scientific, Waltham, MA).

Liquid chromatography mass spectrometry (LCMS) analysis was obtained using a Nexera XR HPLC system (Shimadzu, Carlsbad, CA), consisting of a degassing unit, quaternary pump, autosampler (maintained at 15°C), and column oven (maintained at 40°C) upstream of a Sciex TripleTOF 5600 mass spectrometer (Concord, ON). Chromatographic separation was achieved using an Acquity UPLC BEH C_18_ column (2.1 × 100 mm, 1.7 µm, Waters, Milford, MA) at a flow rate of 0.2 mL/min. The solvents used were water (solvent A) and acetonitrile (solvent B). The column was equilibrated with 95% A/5% B. Upon injection, the mobile phase composition was maintained for 0.5 minutes followed by changing the mobile phase to 0% A/100% B over 19.5 minutes using a linear gradient. The mobile phase was held at 0% A/100% B for 10 minutes. The mobile phase was then changed back to 95% A/5% B, and the column equilibrated for 5 minutes prior to the next injection. The Sciex TripleTOF mass spectrometer was equipped with a DuoSpray Ion Source, and data were collected using the following parameters: negative polarity, nebulizer gas (gas 1) = 35 psi, heater gas (gas 2) = 35 psi, curtain gas = 30 psi, source temperature = 400°C, ion spray voltage floating = −4,500 V, declustering potential = −40 V, collision energy = −5 eV, accumulation time = 0.099982 seconds, and mass range = 100–1,250 m/z. The mass spectrometer was operated using Analyst TF 1.8 software (Sciex, Concord, ON), and the data were processed using PeakView 2.2 software (Concord, ON).

### Construction of reporter constructs

To measure the transcription of *pgnD*, a 326 bp DNA fragment containing the *pgnD* promoter region was amplified from the Pf-5 genome using oligonucleotide pair PFL0260_F2/PFL0260_R2 (Table 1), digested with *Eco*RI and *Hin*dIII, and ligated into pPROBE-NT to generate reporter construct p*pgnD*_promoter_:*gfp*, which contains the *pgnD*_promoter_:*gfp* transcription fusion. Similarly, a 552 bp DNA fragment containing the *pgnC* promoter was amplified using oligonucleotide pair PFL0260_R4 and PFL_0260 UpHindIII, digested with *Eco*RI and *Hin*dIII, and ligated into pPROBE-NT to generate reporter construct p*pgnC*_promoter_:*gfp*, which contains the *pgnC*_promoter_:*gfp* transcriptional fusion. Finally, a 1,342 bp fragment containing *pngC* and promoter of *pngD* amplified from the Pf-5 genome using oligonucleotide pair PFL0260_F2 and PFL0260_R3, digested with *Eco*RI and *Xba*I, and ligated into pPROBE-NT to generate reporter construct p*pgnC-pgnD*_promoter_:*gfp*, which contains the *pgnC-pgnD*_promoter_:*gfp* transcriptional fusion.

To measure the translational expression of the *pgnC* gene, the reporter constructed pPgnC_translation_:*rfp* was made via assembly of three DNA fragments: a 386 bp DNA fragment containing the *pgnC* promoter, the 5′ untranslated region, and the first 10 codons of *pgnC*, amplified using primers RFP_0260_OVLP and GFP_2061_OVLP; a 705 bp DNA fragment containing a promoterless *rfp* gene cloned from pBbE1a using primers RFP_F1 and RFP_R1; and a 6.8 kb DNA fragment containing the pPROBE-NT backbone using primers P35_RFP and P34_0261. The cloned partial *pgnC* gene was fused in-frame with the *rfp* gene to make the translational reporter construct pPgnC_translation_:*rfp*.

The generated reporter constructs were confirmed by Sanger-sequencing analysis and transformed into Pf-5 derivatives via electroporation (1.8 kV, 5 ms) to make the reporter strains.

### Measuring GFP and RFP activities of the reporter strains

Green fluorescence protein (GFP) and red fluorescence protein (RFP) activities of the reporter strains were measured by following our previous method with modifications (47). Pf-5 strains containing reporter constructs were cultured overnight on NBGly plus kanamycin (50 µg/mL) at 28°C. The cells were washed once into 1 mL fresh NBGly plus kanamycin to have an OD_600_ of 1.0 and then used to inoculate 200 µL NBGly plus kanamycin to obtain a start OD_600_ of 0.01. Each strain was grown in three wells of a 96-well plate, which was incubated at 28°C with shaking at 180 rpm in a SPARK multimode Microplate Reader (TECAN, Switzerland). Bacterial growth was monitored by measuring the OD_600_. The GFP activity of bacteria was monitored by measuring emission at 535 nm with an excitation at 485 nm. The RFP activity of bacteria was monitored by measuring emission at 611 nm with an excitation at 568 nm. The fluorescence activity was corrected by subtracting the fluorescence background emitted by the control strain, which contains an empty vector of the reporter construct. Each reporter strain was evaluated with at least three replicates, and the experiment was repeated three times.

### Overexpression and purification of His-tagged RsmE protein

To overexpress and purify the RsmE protein, a 223 bp DNA fragment containing the *rsmE* gene was amplified from the Pf-5 genome using primers PFL2095_F1 and PFL2095_R1 (Table 1). Simultaneously, a 5.3 kb DNA fragment of the pET28a expression vector was amplified using the primers 28aPFL2095_F1 and 28aPFL2095_R1 (Table 1). These two PCR products were assembled using a NEBuilder HiFi DNA Assembly kit (NEB, USA) to generate the expression construct pET28a-RsmE, which contains the *rsmE* gene fused with a 6 × His tag at the C-terminus. The construct was transformed into *Escherichia coli* BL21(DE3), and the cells were cultured in a lysogeny broth medium containing 50 µg/mL kanamycin at 37°C. To induce protein expression, 1 mM isopropyl-β-D-thiogalactopyranoside (IPTG) was added into the culture when cell density reached anOD_600_ of 0.6. The cells were then incubated overnight at 37°C. After incubation, the cells were harvested by centrifugation, washed, and resuspended in binding buffer (20 mM sodium phosphate, 300 mM NaCl, and pH 7.4). The cells were lysed by sonication using a Sonifier SFX250 (Branson, USA). Cell debris was removed by centrifugation at 15,000 rpm for 30 minutes at 4°C.

His-tagged RsmE protein was purified using NEBExpress Ni Resin (NEB, USA) following the manufacturer’s instructions. Briefly, the supernatant containing the His-tagged protein was incubated with Ni resin pre-equilibrated with a binding buffer for 15 minutes. Unbound proteins were removed by washing the resin three times with wash buffer (20 mM sodium phosphate, 300 mM NaCl, 5 mM imidazole, and pH 7.4). The His-tagged RsmE protein was eluted with 2 mL of elution buffer (20 mM sodium phosphate, 300 mM NaCl, 500 mM imidazole, and pH 7.4) and analyzed by SDS-PAGE. The eluted protein was dialyzed against a buffer lacking imidazole and concentrated using Macrosep Advance Centrifugal Devices (Pall Life Sciences, USA) with a 3 kDa molecular weight cutoff. The protein concentration was measured at A280 using a NanoDrop ND‐1000 spectrophotometer (Thermo Fisher Scientific, USA).

### Synthesis of the *pgnC* leader mRNA

To obtain the DNA of the upstream region of *pgnC*, PCR amplification was performed using the forward primer pgnC_UTR_F, which contains the T7 RNA polymerase promoter, and the reverse primer pgnC_UTR_R, with the construct pME6010-*pgnC* as the template. This resulted in a 156 bp PCR product. The mRNA was then transcribed *in vitro* from the PCR product using the HiScribe T7 High Yield RNA Synthesis Kit (NEB, USA). Transcription reactions were set up with 1 µg of purified PCR product, RNA NTPs (7.5 mM each of ATP, CTP, and GTP, 5 mM UTP, and 2.5 mM Biotin-16-UTP [Roche, Sigma]), 5 mM dithiothreitol (DTT), T7 RNA Polymerase Mix, and the provided reaction buffer. The transcription reaction was incubated at 37°C overnight. Following transcription, the RNA was purified using the Monarch RNA Cleanup Kit (NEB, USA) according to the manufacturer’s protocol. The purified RNA was quantified by measuring absorbance at 260 nm, confirmed as a single band by electrophoresis on 1.5% agarose gel, and stored at −80°C until further use.

### AlphaScreen assay

The interaction between *pgnC* leader mRNA and RsmE was detected by AlphaScreen assay modified from our previous method (48). Briefly, the assays were conducted in an assay buffer consisting of 30 mM HEPES (pH 7.5), 100 mM KCl, 40 mM NaCl, 10 mM ammonium acetate, 10 mM guanidinium hydrochloride, 2 mM MgCl2, 0.5 mM EDTA, 3% DMSO, and 0.01% NP-40 using an AlphaScreen histidine (nickel chelate) detection kit (Perkin Elmer). All reactions were performed in 384-well white polystyrene plates (Corning) with a final volume of 30 µL/well. In each well, His-tagged RsmE protein and biotinylated *pgnC* leader mRNA (15 µL total) were added and incubated for 30 minutes at room temperature. Following this, 10 µg/mL of acceptor beads was added to each well, and the mixture was incubated for an additional 30 minutes. Subsequently, 10 µg/mL of donor beads was added to each well and incubated for another 30 minutes. The AlphaScreen signal was measured using a Spark Multimode Microplate Reader (Tecan) equipped with the HTS AlphaScreen module. All reactions were performed in triplicate to ensure reproducibility.

### Statistical analysis

Statistical analyses were performed by Student *t*-test (SPSS Inc., Chicago, IL). All replicates in this study were biological replicates. All data are presented as mean ± SD.

## RESULTS

### Polyynes of Pf-5 are toxic to *P. syringae* pv. *tomato* DC3000

*P. syringae* pv. *tomato* DC3000, a Gram-negative bacterial plant pathogen, was used as an indicator bacterium in the inhibition assays because our previous study showed that DC3000 could be inhibited by Pf-5 on culture plates (28). Consistent with the previous report, wild-type Pf-5 inhibited the growth of DC3000 on NAGly medium (Fig. 1A), suggesting that Pf-5 produced an antibacterial metabolite that is toxic to DC3000. To pinpoint the toxic metabolite, Pf-5 mutants that lack the production of different antibiotics were used in the inhibition assays.

Compared to the wild-type strain Pf-5, a similar inhibition of DC3000 was observed by using a mutant ∆*prnC*∆*phlD*∆*rzxB*∆*pltA*∆*hcnB*∆*ofaA* (called sixfold mutant hereinafter; Fig. 1A), which does not produce any of the six antibiotics (pyrrolnitrin, 2,4-diacetyalphloroglucinol, rhizoxin, pyoluteorin, hydrogen cyanine, or orfamide A) because of mutation of the biosynthetic genes (Table 1). This result suggests that other antibiotic(s) produced by Pf-5 is(are) involved in the inhibition. Previously, we found that Pf-5 produces toxoflavin, which inhibited DC3000 on KB medium supplemented with FeCl_3_ (28). However, mutation of the toxoflavin biosynthetic gene *toxB* in the sixfold mutant background (the resulting mutant was called sevenfold-A mutant hereinafter) inhibited DC3000 on NAGly (Fig. 1A), suggesting that toxoflavin did not contribute to the inhibition of DC3000 on NAGly. We then deleted the polyyne biosynthetic gene *pgnE* (Fig. 1B) in the sixfold mutant background. The generated mutant, ∆*prnC*∆*phlD*∆*rzxB*∆*pltA*∆*hcnB*∆*ofaA*∆*pgnE* (called sevenfold-B mutant hereinafter), did not inhibit DC3000 (Fig. 1A). Furthermore, the inhibition was restored by introducing a wild-type *pgnE*, carried on a construct pME6010-*pgnE*, into the sevenfold-B mutant (Fig. 1C).

**Fig 2:**
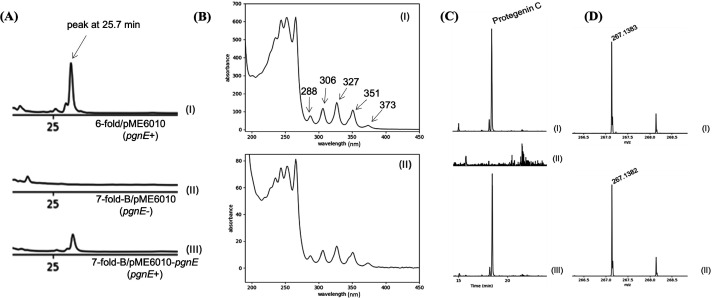
Chemical analysis of Pf-5’s metabolites on NAGly medium by HPLC (A and B) and LCMS (C and D). (A) HPLC chromatograms (254 nm) of metabolite extracts of (I) sixfold/pME6010, (II) sevenfold-B/pME6010, and (III) sevenfold-B/pME6010-*pgnE*. (B) UV spectra of the peak eluting at 25.2 minutes in the extracts of (I) and (III). (C) Extracted ion chromatogram (267.135–267.145) for protegenin C in the extracts of (I), (II), and (III). (D) Mass spectrum of the major protegenin C peak (calc. [M-H]^−^ = 267.1391) as seen in the chromatogram of (I) (obs. [M-H]^−^ = 267.1383, 2.99 ppm error) and (II) (obs. [M-H]^−^ = 267.1382, 3.37 ppm error).

To evaluate the presence of polyyne in the cultures of *P. protegens* Pf-5 strains, metabolites produced by the strains on NAGly medium were extracted and subjected to HPLC and LCMS analyses. The HPLC chromatograms of the sixfold/pME6010 and sevenfold-B/pME6010-pgnE extracts show the presence of a peak eluting at 25.7 minutes, while this peak is absent in the extract of the sevenfold-B/pME6010 mutant (Fig. 2A). The UV spectrum of this peak shows that it has multiple relative maxima at 288 nm, 306 nm, 327 nm, 351 nm, and 373 nm (Fig. 2B). These relative maxima are consistent with the reported UV absorbances for the protegenins (20). The presence of protegenin A, C, and D in the extracts of sixfold/pME6010 and sevenfold-B/pME6010-*pgnE*, but not the sevenfold-B/pME6010, was further confirmed by LCMS analysis (Fig. 2C and D; Fig. S1 to S3; Table S1).

These results indicate that the polyynes of Pf-5 exhibit antibacterial activity against DC3000. Additionally, these data show that the sixfold mutant, but not the sevenfold-B mutant, produced polyynes on NAGly medium, thus validating that these two Pf-5 derivatives and the current inhibition assay method could be used to test the antibacterial activity of the polyynes produced by Pf-5.

### Polyynes of Pf-5 have a broad spectrum of antibacterial activity

**Fig 3:**
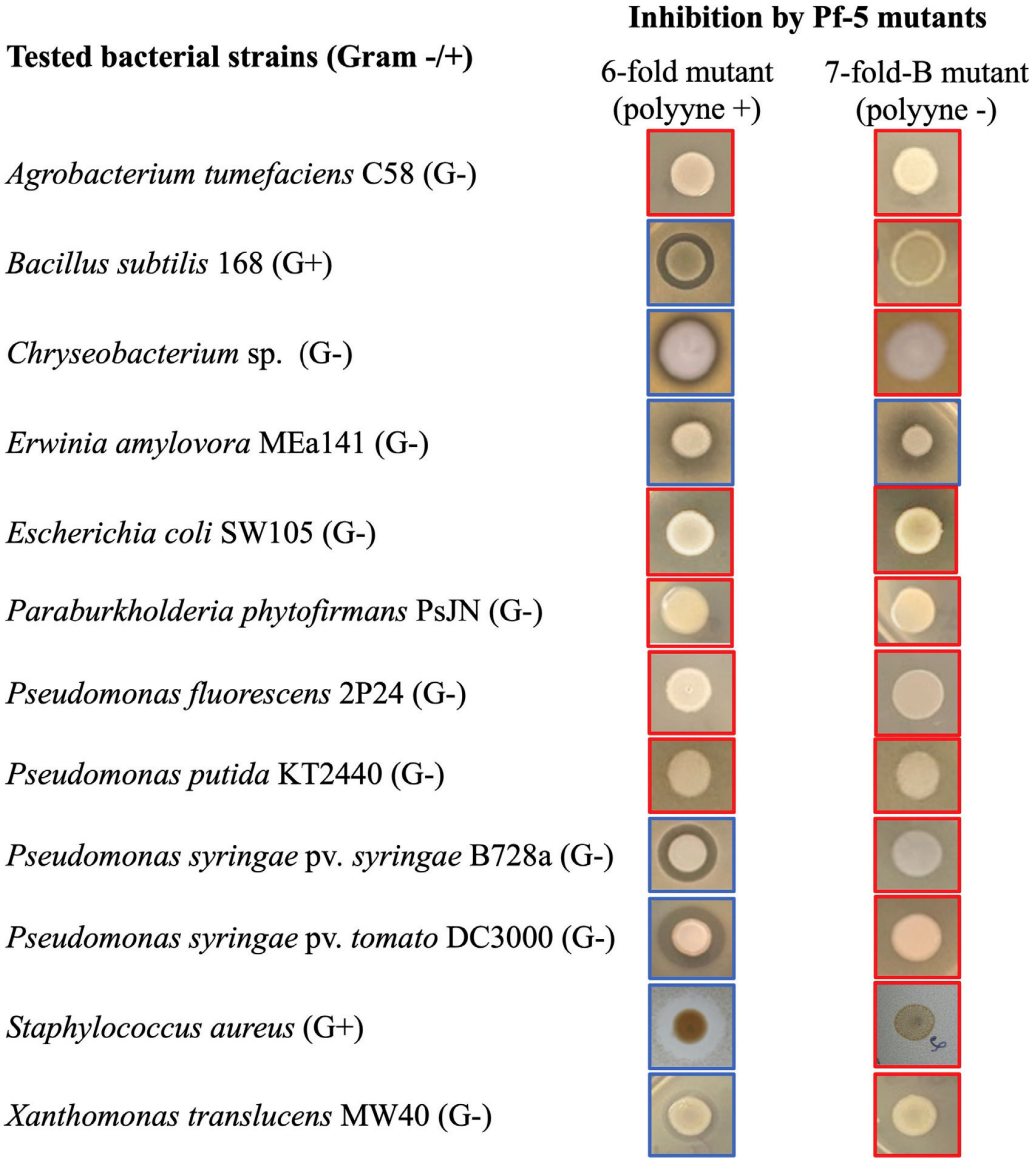
Antibacterial activity of Pf-5-produced polyyne. The assays were conducted by mixing the tested bacterial strains in NAGly and then challenged by Pf-5 derivatives, including the polyyne-producing sixfold mutant and the polyyne nonproducing sevenfold-B mutant. G+: Gram-positive, G−: Gram-negative. Red and yellow frames of the photo indicate inhibition and no inhibition, respectively, of the tested bacteria. The experiment was repeated at least two times independently.

To understand if the polyynes of Pf-5 have a broad antibacterial activity, 11 different bacterial strains (in addition to DC3000, which has been tested above), including two Gram-positive bacteria, were tested for inhibition by the polyyne-producing sixfold mutant and the nonproducing sevenfold-B mutant. As shown in Fig. 3, five tested bacterial strains, including *Bacillus subtilis* 168, *Chryseobacterium* sp., *P. syringae* pv. *syringae* B728a, *Staphylococcus aureus*, and *Xanthomonas translucens* MW40, were inhibited by the polyyne-producing strain but not the nonproducing strain. This result indicates that these five strains are sensitive to the polyynes of Pf-5. Another five tested strains, including *Agrobacterium tumefaciens* C58, *E. coli* SW105, *Paraburkholderia phytofirmans* PsJN, *Pseudomonas fluorescens* 2P24, and *Pseudomonas putida* KT2440, were not inhibited by either the polyyne-producing strain or the nonproducing strain. This result indicates that these five strains are tolerant of the polyynes produced by Pf-5. One strain, *Erwinia amylovora* MEa141, was inhibited by both the polyyne-producing and nonproducing strains, indicating that the inhibition does not require the polyynes. Overall, *P. protegens*-derived polyynes have a broad antibacterial activity and can be toxic to both Gram-negative and Gram-positive bacteria.

### Polyyne biosynthesis is positively regulated by pathway-specific regulator PgnC

**Fig 4:**
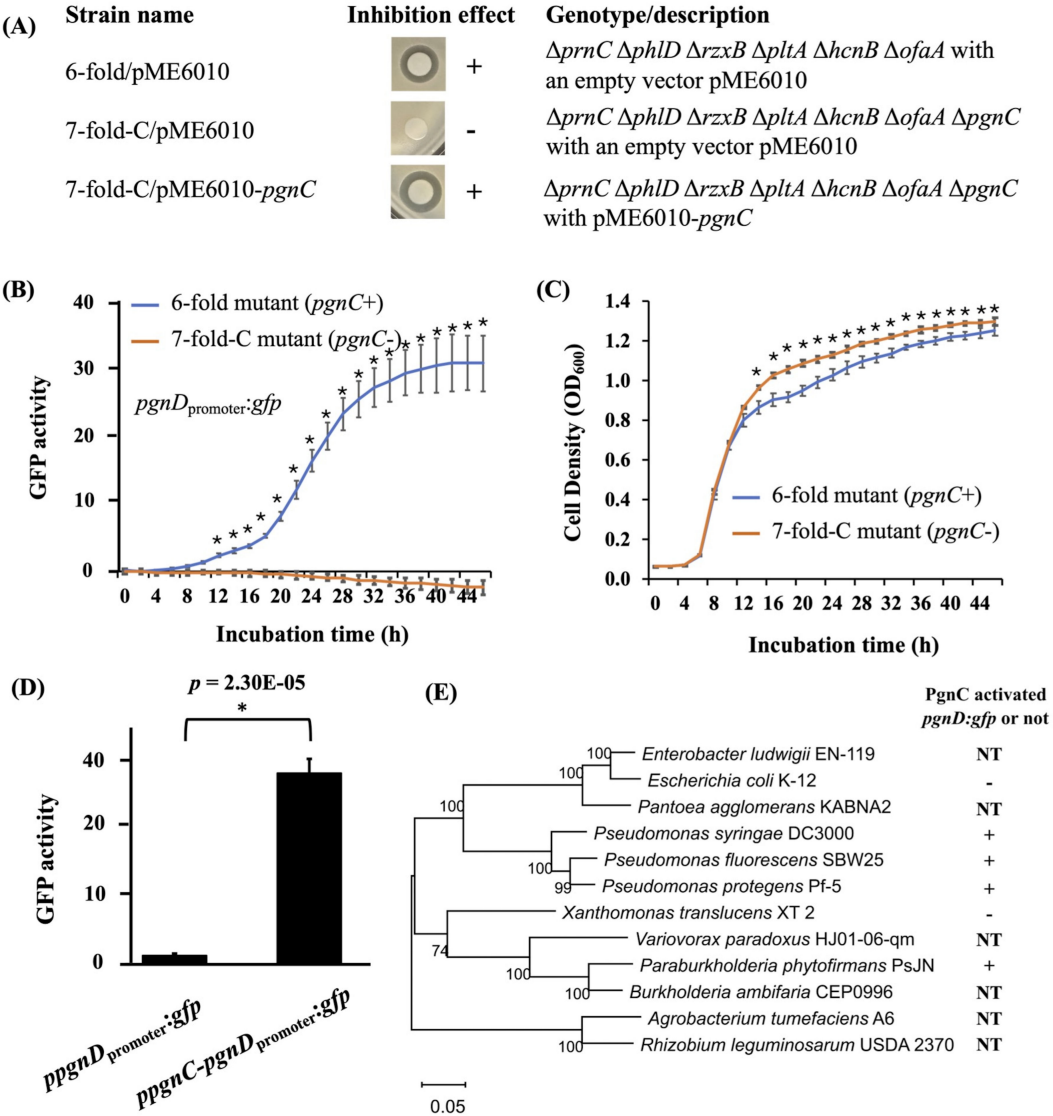
Expression of polyyne biosynthetic genes is controlled by PgnC. (A) Mutation of *pgnC* abolished the inhibition of Pf-5 to the growth of *P. syringae* DC3000 on NAGly. (B) Expression of the *pgnD*_promoter_:*gfp* transcriptional reporter fusion in the sixfold mutant and the sevenfold-C mutant in NBGly. (C) Growth curves of the sixfold mutant and the sevenfold-C mutant in NBGly. (D) Expression of the *pgnD*_promoter_:*gfp* reporter fusion without *pgnC* (carried by the *ppgnD*_promoter_:*gfp* construct) or with *pgnC* (carried by the *ppgnC-pgnD*_promoter_:*gfp* construct) in *P. fluorescens* SBW25 (D) and other tested bacterial strains (E) in NBGly. * Indicates significant difference as determined by the Student *t*-test. The *P* value is either less than 0.01 or included. “+” and “−” Indicate activation and no activation of *pgnD*_promoter_:*gfp*, respectively, by the regulator PgnC. NT: not tested. Data are means ± SD of three replications of each treatment. All the experiments were repeated at least three times, which generated similar results.

A regulatory gene *pgnC* is located in the polyyne biosynthetic gene cluster of Pf-5 (Fig. 1B). However, its role in polyyne biosynthesis remains unknown. To test if *pgnC* plays a role in polyyne production, the gene was deleted in the ∆*prnC*∆*phlD*∆*rzxB*∆*pltA*∆*hcnB*∆*ofaA* sixfold mutant. The generated mutant ∆*prnC*∆*phlD*∆*rzxB*∆*pltA*∆*hcnB*∆*ofaA*∆*pgnC* (called sevenfold-C mutant hereafter) did not inhibit DC3000 (Fig. 4A), indicating *pgnC* is required for polyyne biosynthesis. The role of *pgnC* in polyyne biosynthesis was confirmed by the result that introducing a wild-type *pgnC* gene in the sevenfold-C mutant using the complementation construct pME6010-*pgnC* restored the inhibition of DC3000 on NAGly plates (Fig. 4A).

Gene annotation suggests that *pgnC* encodes a putative AraC-type transcriptional regulator (49). We hypothesized that PgnC positively regulates the expression of polyyne biosynthetic genes. To test this hypothesis, we made a transcriptional reporter construct that contains the promoter region of *pgnD*, the first gene of the polyyne biosynthetic gene cluster (Fig. 1B), fused with a promoterless *gfp* gene. The resultant reporter construct, p*pgnD*_promoter_:*gfp*, was transformed into the sixfold mutant and the sevenfold-C mutant. We found that mutation of *pgnC* abolished the GFP activity of the reporter strain (Fig. 4B). Reduced GFP activity could be caused by reduced growth of the *pgnC* mutant, but this possibility was rejected because the mutation of *pgnC* slightly increased the bacterial growth at late exponential and early stationary stages (Fig. 4C). These results indicate that *pgnC* is required to activate the promoter of *pgnD*, thus supporting our hypothesis that PgnC positively regulates the transcription of the polyyne biosynthetic genes.

The intergenic region between *pgnC* and *pgnD* contains an inverted repeat (Fig. S4), which is common for binding sites of the AraC family regulators (50, 51). To test if PgnC directly binds to the intergenic region, exhaustive efforts were made to purify PgnC but failed due to the insolubility of the protein. As an alternative approach, we tested the expression of *pgnD*_promoter_:*gfp* in *P. fluorescens* SBW25, which does not contain the polyyne biosynthetic genes. We found that strain SBW25 containing the reporter construct p*pgnD*_promoter_:*gfp* had a basal expression level of GFP activity. However, SBW25 containing p*pgnC-pgnD*_promoter_:*gfp*, which includes the *pgnC* regulatory gene, had a very strong GFP activity (Fig. 4D). This result indicates that PgnC activated the *pgnD* promoter heterogeneously in SBW25 and supports the notion that PgnC likely directly activates the transcription of polyyne biosynthetic genes in Pf-5.

Moreover, we found that PgnC activated the expression of *pgnD*_promoter_:*gfp* heterogeneously in *P. syringae* pv. *tomato* DC3000 and *P. phytofirmans* PsJN, further supporting a direct role of PgnC in the activation of *pgnD* expression. However, PgnC did not activate the expression of *pgnD*_promoter_:*gfp* in *E. coli* or *X. translucens* (Fig. 4E), indicating that the *pgnD* promoter does not function properly in these two strains.

Overall, these results support a direct role of PgnC in the regulation of polyyne production, which acts as a pathway-specific regulator directly activating the transcription of polyyne biosynthetic genes.

### GacA controls polyyne production by regulating the expression of *pgnC*

**Fig 5:**
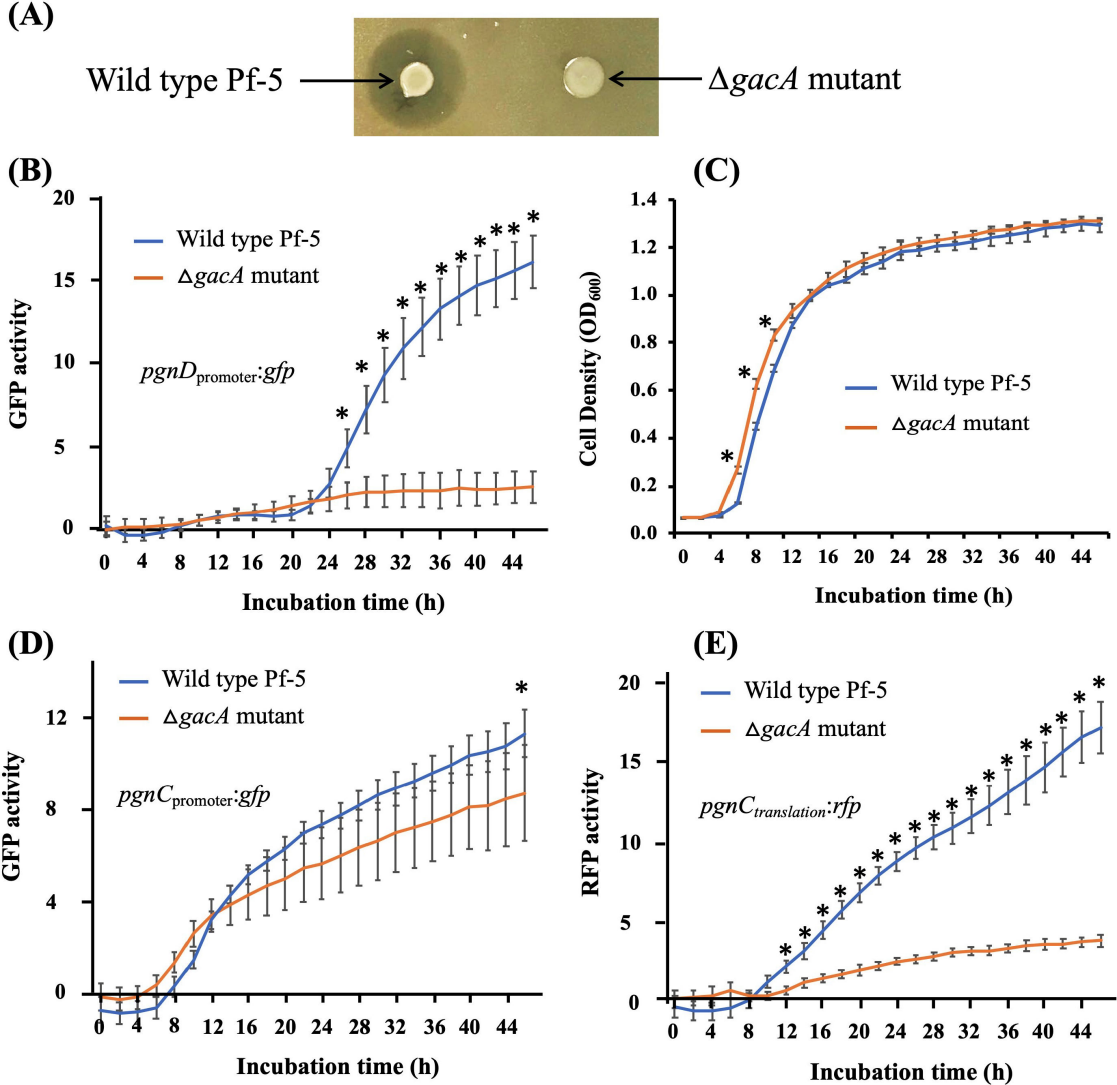
Expression of polyyne biosynthesis genes is controlled by the GacA global regulator. (A) The Δ*gacA* mutant did not inhibit the growth of *P. syringae* DC3000 on NAGly. (B) Expression of the *pgnD*_promoter_:*gfp* transcriptional reporter fusion in the wild-type Pf-5 and Δ*gacA* mutant in NBGly. (C) Growth curves of the wild-type Pf-5 and Δ*gacA* mutant in NBGly. (D) Expression of the *pgnC*_promoter_:*gfp* transcriptional reporter fusion in the wild-type Pf-5 and Δ*gacA* mutant in NBGly. (E) Expression of the *pgnC_t_*_ranslation_:*rfp* translational reporter fusion in the wild-type Pf-5 and Δ*gacA* mutant in NBGly. Data are means ± SD of three replications of each treatment. * Indicates significant difference as determined by the Student *t*-test (*P* < 0.01). All the experiments were repeated at least three times, generating similar results.

In addition to pathway-specific regulation, the GacS/GacA global regulatory system controls many pathways of secondary metabolism in Pf-5 (52). As expected, a mutation of *gacA*, which encodes a regulatory protein of the GacS/GacA regulatory system, abolished the inhibition of Pf-5 to DC3000 (Fig. 5A), suggesting that GacA positively regulates the polyyne biosynthesis of Pf-5.

To understand the mechanism of how GacA regulates polyyne biosynthesis, we tested the expression of the polyyne gene in wild-type Pf-5 and ∆*gacA* mutants using the reporter construct p*pgnD*_promoter_:*gfp*. Compared to the wild-type strain, the ∆*gacA* mutant had a significantly lower GFP level of the reporter construct (Fig. 5B), indicating that GacA positively regulates the transcription of the polyyne genes. Mutation of *gacA* increased bacterial growth at the exponential stage (Fig. 5C), which is consistent with our previous report (29).

Based on the result that PgnC directly regulates the transcription of the polyyne genes, we hypothesized that GacA induces the expression of PgnC, which in turn activates the polyyne biosynthesis. To test this hypothesis, we made a transcriptional reporter construct that contains the promoter region of *pgnC* fused with a promoterless *gfp* gene. The GFP activity of the resultant reporter construct, p*pgnC*_promoter_:*gfp*, was measured in the wild-type Pf-5 and the ∆*gacA* mutant. We found that, compared to the wild type, the *∆gacA* mutant had a slightly decreased GFP activity of the reporter construct (Fig. 5D), suggesting that GacA positively regulates the transcription of *pgnC*.

However, it is noteworthy that the ∆*gacA* mutant still expressed a decent level of the transcription of *pgnC* (Fig. 5D). This result cannot fully explain the abolished inhibition of DC3000 by the ∆*gacA* mutant, because partial *pgnC* transcription in the ∆*gacA* mutant could lead to lower expression of the polyyne biosynthetic genes and produce a partial level of the antibiotic that would reduce, but not abolish, the inhibition of DC3000. We hypothesized that GacA also regulates the translation of *pgnC*. To test this hypothesis, we made a translational reporter construct that contains the *pgnC* promoter, the 5′ untranslated region, and the first 10 codons of *pgnC* fused in-frame with an *rfp* gene. The resultant construct pPgnC_translation_:*rfp* was transformed into Pf-5 and its ∆*gacA* mutant. The RFP activity of the reporter strains was measured. Results showed that the expression of PgnC_translation_:*rfp* is reduced in the ∆*gacA* mutant compared to the wild type (Fig. 5E).

Together, these results suggest that GacA positively regulates the transcription and translation of PgnC. The regulation of PgnC expression by GacA is primarily at the translational level.

### RsmE binds directly to the *pgnC* leader mRNA

**Fig 6:**
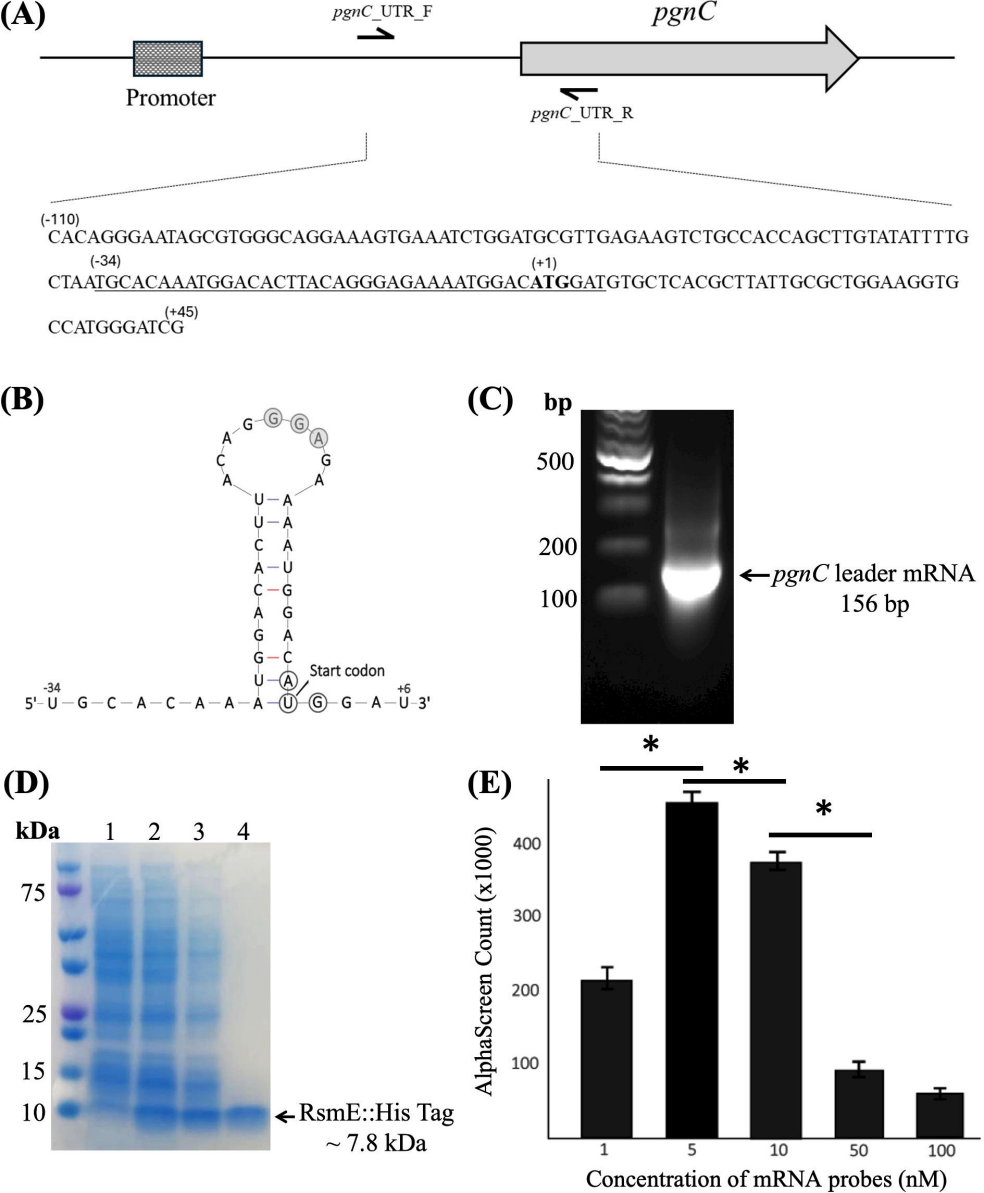
RsmE interacts with the *pgn*C leader mRNA. (A) Schematic representation of the upstream region of *pgn*C. The two black arrows indicate PCR primers to generate a 156 bp mRNA fragment that was labeled with biotin and used in the AlphaScreen assay. (B) Predicted secondary structure of the *pgnC* leader mRNA (sequences were shown in [A] with an underline) generated by Mfold. The putative RsmE binding site is indicated by gray circles. The start codon of *pgnC* is also indicated. (C) Evaluation of the synthesized *pgnC* leader mRNA via agarose gel electrophoresis analysis. (D) Evaluation of the purified RsmE::His protein via SDS-PAGE gel electrophoresis analysis. Lane 1: protein ladder; 2: protein profile of *E. coli* cells without IPTG induction; 3: protein profile of *E. coli* cells after IPTG induction; 4: soluble proteins from the lane 3 sample; 5: purified recombinant His-tag RsmE. (E) AlphaScreen assay shows the interaction between RsmE::His-tag and biotinylated *pgn*C leader mRNA. RsmE::His-tag (1 nM) was amended with different concentrations (1, 5, 10, 50, and 100 nM) of the *pgnC* leader mRNA. Data are means ± SD of three replications of each treatment. * Indicates significant difference as determined by the Student *t*-test (*P* < 0.01). The experiment was repeated at least three times, generating similar results.

GacA is known to induce the expression of regulatory RNAs that sequester the RNA-binding proteins RsmA and RsmE, which block the translation of target mRNAs (53). Sequence analysis predicts that the *pgnC* leader mRNA forms a stem-loop structure with a potential RsmA/RsmE binding motif “GGA” in the loop (Fig. 6A and B). We hypothesized that RsmA and RsmE bind directly to the *pgnC* leader mRNA and inhibit its translation. To test this hypothesis, we purified the RsmE protein and synthesized a 156 bp *pgnC* leader mRNA, which has 110 bp upstream and 45 bp downstream of the *pgnC* start codon (Fig. 6C and D). The interaction between the RsmE protein and the *pgnC* leader mRNA was assessed by AlphaScreen analysis. The result shows that the signal of the AlphaScreen counts enhanced with the increased amount of the *pgnC* leader mRNA probe added in the RsmE protein solution (Fig. 6E), suggesting a direct binding of the RsmE protein to the *pgnC* leader mRNA. The signal declined when a higher amount of the mRNA probe (≥10 nM) was added to the protein solution, which is typical in an AlphaScreen assay and likely caused by competition among probe molecules at saturating concentrations that interfere with bead pairing (54).

## DISCUSSION

**Fig 7:**
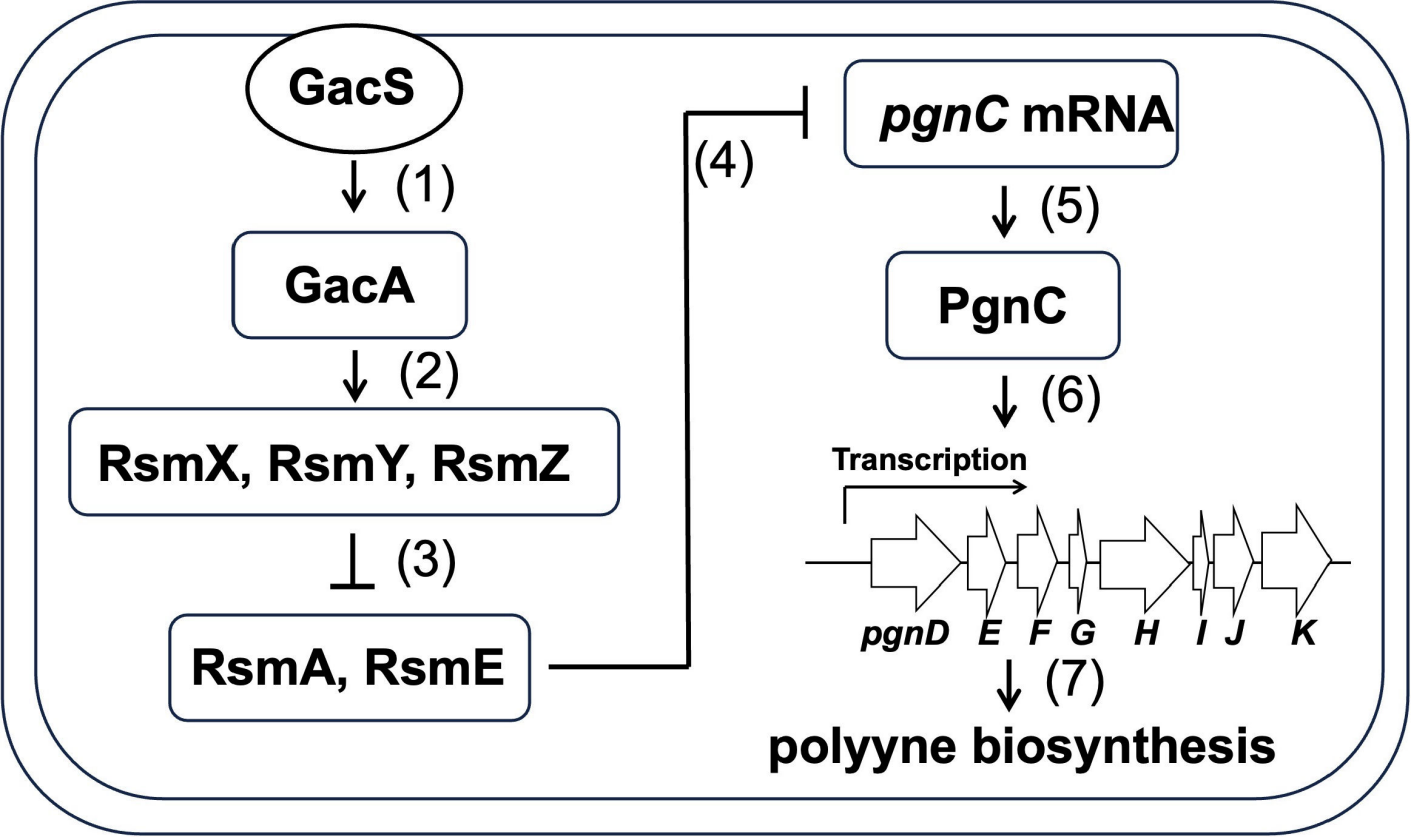
Model for regulation of polyyne biosynthesis by PgnC and GacA in *P. protegens* Pf-5 (1). GacS sensor activates GacA through phosphorylation (2); phosphorylated GacA induces the expression of small regulatory RNAs RsmX, RsmY, and RsmZ (3); the small RNAs sequester the RNA-binding proteins RsmA and RsmE (4); RsmE binds *pgnC* leader mRNA and blocks its translation (5); *pgnC* mRNA is translated into PgnC due to the sequestration of RsmE by RsmX, RsmY, and RsmZ (6). PgnC directly activates the transcription of *pgn* genes, which leads to (7) the biosynthesis of polyyne compounds. The proposed regulation pathway is supported by the results of this study and previous reports (24, 25).

This study reports that polyynes produced by *P. protegens* Pf-5 can have a broad spectrum of antibacterial activity against Gram-positive bacteria (e.g., *B. subtilis* and *S. aureus*) and Gram-negative bacteria (e.g., *Chryseobacterium* sp., *P. syringae* pv. *syringae*, *P. syringae* pv. *tomato*, and *X. translucens*). The expression of the polyyne biosynthetic genes of Pf-5 is regulated by both a pathway-specific regulator and a global regulator, as shown in a simplified model (Fig. 7). The polyyne biosynthesis is positively regulated by the pathway-specific regulator PgnC that is required to activate the transcription of the polyyne biosynthetic genes. The expression of PgnC is controlled by the global regulator GacA, which positively regulates the translation of PgnC and activates the polyyne biosynthetic pathway.

### PgnC directly activates the transcription of polyyne biosynthetic genes in Pf-5

Binding assays to test the interaction between PgnC protein and the *pgnD* promoter DNA failed due to the insolubility of recombinant His-tagged PgnC. However, PgnC strongly activated the expression of the *pgnD* promoter heterogeneously in *P. syringae* DC3000*, P. fluorescens* SBW25, and *P. phytofirmans* PsJN (Fig. 4D and E). These results support our notion that PgnC directly activates the *pgnD* promoter in Pf-5. It is interesting that some of the tested strains did not support the PgnC-mediated activation of the *pgnD* promoter. For example, PgnC failed to activate the expression of *pgnD*_promoter_:*gfp* in *E. coli* or *X. translucens* (Fig. 4E), indicating that the *pgnD* promoter may not function properly in these bacteria. A helping factor (such as a sigma factor), which is present in Pf-5 and closely related bacteria, is likely required to activate the expression of the polyyne biosynthetic genes. It is known that antibiotic production can be influenced by sigma factors (55, 56). The sigma factor(s) may be involved in the transcription of either *pgnD* or *pgnC*, or both. Alternatively, factors other than the sigma factor can also contribute to the PgnC-mediated activation of the *pgnD* promoter. For example, a signal compound may serve as an inducer of PgnC, which then activates the expression of *pgnD*. PgnC is a member of the AraC family of transcriptional regulators. AraC of *E. coli* has a dual activity as a transcriptional repressor and activator depending on the presence of the inducer compound arabinose (51). To date, a signaling compound or additional regulatory factor influencing PgnC has not been identified in strains of *Pseudomonas* and *Burkholderia* that produce polyynes. Nevertheless, these potential additional factors are likely conserved in *Pseudomonas* and *Burkholderia*, which is not surprising because polyyne derivatives have been found in these two groups of bacteria (9, 17, 18).

### Pf-5 may coordinate the polyyne biosynthesis with other antibiotic biosynthetic pathways

Compared to the wild-type Pf-5, the sixfold mutant expressed *pgnD*_promoter_:*gfp* reporter fusion at a much higher level and earlier growth stage (Fig. 4B and 5B). This result indicates that polyyne biosynthesis is negatively influenced by the production of one or more of the other six antibiotics and implies a possible metabolic coordination. Coordination between different metabolite biosynthetic pathways is common in bacteria (57–59). This coordination can be due to the coordinated regulation of biosynthetic gene clusters, as occurs between the pyoluteorin and 2,4-diacetylphloroglucinol gene clusters of Pf-5 (60, 61). Alternatively, different antibiotic pathways may influence each other through competition for shared biosynthetic substrates and limited cellular resources such as precursors generated from primary metabolism (37, 62). Antibiotic biosynthesis can also be energetically costly (29). It is possible that the shutdown of the six antibiotic pathways resulted in an accumulation of the substrate(s), precursor(s), or energy that, in turn, led to an enhanced level of polyyne biosynthesis. Understanding the coordination between different antibiotic pathways can help us activate pathways that are not expressed under normal laboratory conditions and manipulate antibiotic production.

### The polyynes produced by Pf-5 can have a broad spectrum of antibacterial activity in both Gram-negative and Gram-positive bacteria

The *P. protegens*-derived polyynes are known to have antialgal (17), antifungal, and anti-oomycete activities (20). Our results extended the antimicrobial spectrum of *P. protegens*-derived polyynes to Gram-positive and Gram-negative bacteria (Fig. 3). The bacterial toxicity of polyynes was also reported previously, with polyynes named caryonencins produced by *T. caryophylli* being toxic to *S. aureus*, *B. subtilis*, and *Klebsiella pneumoniae* (12). However, not all the known bacterial polyynes have antibacterial activity. For example, fischerellin A, a polyyne produced by a cyanobacterium *F. muscicola* has antifungal, antialgal, and herbicidal activity but did not inhibit any of the tested bacterial strains including *B. subtilis* and *P. fluorescens* (14, 63).

Variations of polyyne tolerance were observed among the tested Gram-negative bacteria even from different strains of the *Pseudomonas* genus. Interestingly, three of the four sensitive strains are leaf pathogens, including the barley leaf pathogen *X. translucens* MW40 (lab collection, data not shown), the tomato leaf pathogens *P. syringae* pv. *tomato* DC3000, and *P. syringae* pv. *syringae* B728a (42). Four of the five tolerant strains are either root pathogens (*A. tumefaciens* C58 [32]) or beneficial bacteria that were originally isolated from root (*P. phytofirmans* PsJN [34]) or soil (*P. fluorescens* 2P24 and *P. putida* KT2440 [41, 64]). These results suggest that bacterial living habits influence polyyne tolerance, although testing more strains is needed to evaluate this hypothesis. Consistent with this notion, reports have indicated that soil is a major reservoir of antibiotic-resistance genes (65, 66). The genome of *P. putida* KT2440 encodes a large number of multidrug efflux systems and inactivating/modifying enzymes that were predicted to play a role in protecting the bacterium from toxic compounds (67). Self-resistance of polyyne in *Massilia* sp. YMA4, which produces polyynes called massilins, involves an acetyl-CoA acetyltransferase encoded in the polyyne biosynthesis gene cluster (8). No acetyl-CoA acetyltransferase was found in the polyyne biosynthesis gene cluster of *P. protegens* Cab57 (20) or Pf-5 (17, 19), indicating that *P. protegens* may use a different self-resistance mechanism. A potential drug resistance transporter encoding gene PFL_0259 is located next to the transcriptional regulator gene *pgnC* (PFL_0260) in the genome of Pf-5 (49). Future research is needed to test if PFL_0259 plays a role in the self-resistance of polyynes in Pf-5. Massilins inhibited the fungal growth of *Candida albicans* by disrupting its cell membrane integrity and ergosterol biosynthesis (8). Further analysis of the sensitive and tolerant bacterial strains identified in this study may help us find the bacterial target(s) and mode of action of the polyynes produced by *P. protegens*.

The antibacterial activity assay of this study does not provide detailed information on the antibacterial activity of individual polyyne derivatives produced by Pf-5. Three polyyne derivatives including protegenin A, C, and D were found to be produced by Pf-5 in this study (Fig. 2C and D; Fig. S1 and S2) and a previous report (19). Protegenin A, C, and D were detected from Pf-5’s cultures of the polyyne-producing strain but not the polyyne-nonproducing strain used in the antibacterial activity assay (Fig. 2C and D; Fig. S1 and S2). Hence, the results of the current antibacterial activity assay indicate the toxicity of the combined protegenins but not of each individual protegenin. Four polyyne derivatives, protegenin A, B, C, and D, were found to be produced by *P. protegens* Cab57. Among these four protegenins, A and B showed stronger antifungal and anti-oomycete activities than C and D (20). The antibacterial activity of the individual protegenin of Pf-5 was not tested in this work due to the limited availability of the purified compounds. Nevertheless, the results obtained in this study, which used the polyyne-producing strain and nonproducing strain, still provided useful insights into the antimicrobial activity of the polyynes produced by *P. protegens*.

Antibiotics are secondary metabolites that play important roles in the ecological fitness of their producers (68). Understanding their antimicrobial activity can advance the use of these metabolites and the producing bacteria in agriculture and other aspects of our society. For example, researchers found that polyynes of *P. protegens* Cab57 inhibit the Oomycete plant pathogen *Pythium ultimum* and used this bacterium to control damping-off disease of cucumber seedlings caused by *P. ultimum* (20). In this work, we report that polyynes produced by Pf-5 strongly inhibited bacterial leaf pathogens, including *P. syringae* pv. *syringae*, *P. syringae* pv. *tomato*, and *X. translucens* (Fig. 3). It will be interesting to test if Pf-5 and/or its polyynes can control any of the bacterial leaf diseases in the future.

### Conclusion

This study investigated the molecular mechanisms by which bacteria regulate the production of polyyne antibiotics. Results indicate that PgnC, a transcriptional regulator encoded in the polyyne biosynthetic gene cluster of the soil bacterium *P. protegens* Pf-5, directly activates the expression of polyyne biosynthetic genes. Expression of PgnC is induced by the global regulator GacA, which negatively regulates the small regulatory protein RsmE that binds directly to the *pgnC* leader mRNA. These results reveal a regulatory mechanism by which Pf-5 controls polyyne production at both the pathway-specific level and a higher global level. This study also tested the antibacterial activities of polyynes produced by Pf-5 and showed that the polyyne-producing strain, but not the polyyne-nonproducing strain, inhibited a broad spectrum of Gram-negative and Gram-positive bacteria. Overall, this study advanced our understanding of the molecular mechanisms that regulate polyyne production and extended the antimicrobial spectrum of polyynes, which may enhance the opportunity to implement their use in agriculture or medicine.

## Data Availability

The data the support the findings of this study are available upon request.

## SUPPLEMENTAL MATERIAL

Supplemental material aem.02388-24-s0001.docx Table S1; Figures S1 to S4.

ASM does not own the copyrights to Supplemental Material that may be linked to, or accessed through, an article. The authors have granted ASM a non-exclusive, world-wide license to publish the Supplemental Material files. Please contact the corresponding author directly for reuse.
