# Supplementary material for: Polyyne production is regulated by the transcriptional regulators PgnC and GacA in Pseudomonas protegens Pf-5
Source: Appl Environ Microbiol. 2025 Apr 3;91(4):e02388-24. doi: 10.1128/aem.02388-24 (PMC12016544; doi:10.1128/aem.02388-24)
Supplement: Supplemental material — Table S1; Figures S1 to S4. [file aem.02388-24-s0001.docx]

**Polyyne production is regulated by the transcriptional regulators PgnC and GacA in *Pseudomonas protegens* Pf-5**

**Authors:** Chiseche Mwanza^a^, Maria Purnamasari^a^, Daniel Back^b^, Cahya Prihatna^a^, Benjamin Philmus^c^, Khaled H. Almabruk^c^, Taifo Mahmud^c^, Lumeng Ye^d^*, Melvin D. Bolton^b^, Xiaogang Wu^e^, Joyce E. Loper^f^, Qing Yan^a^#

**Table S1**. Peak areas of protegenins A, C, and D in strains 6-fold/pME6010 and 7-fold-B/pME6010-*pgnE* from their respective extracted ion chromatograms.

| **Bacterial strains** | **Peak area** | | |
| --- | --- | --- | --- |
|  | **Protegenin A** | **Protegenin C** | **Protegenin D** |
| 6-fold/pME6010 | 6.67 x 10^5^ | 6.72 x 10^5^ | 1.14 x 10^5^ |
| 7-fold-B/pME6010-*pgnE* | 7.40 x 10^5^ | 5.30 x 10^5^ | 8.64 x 10^4^ |


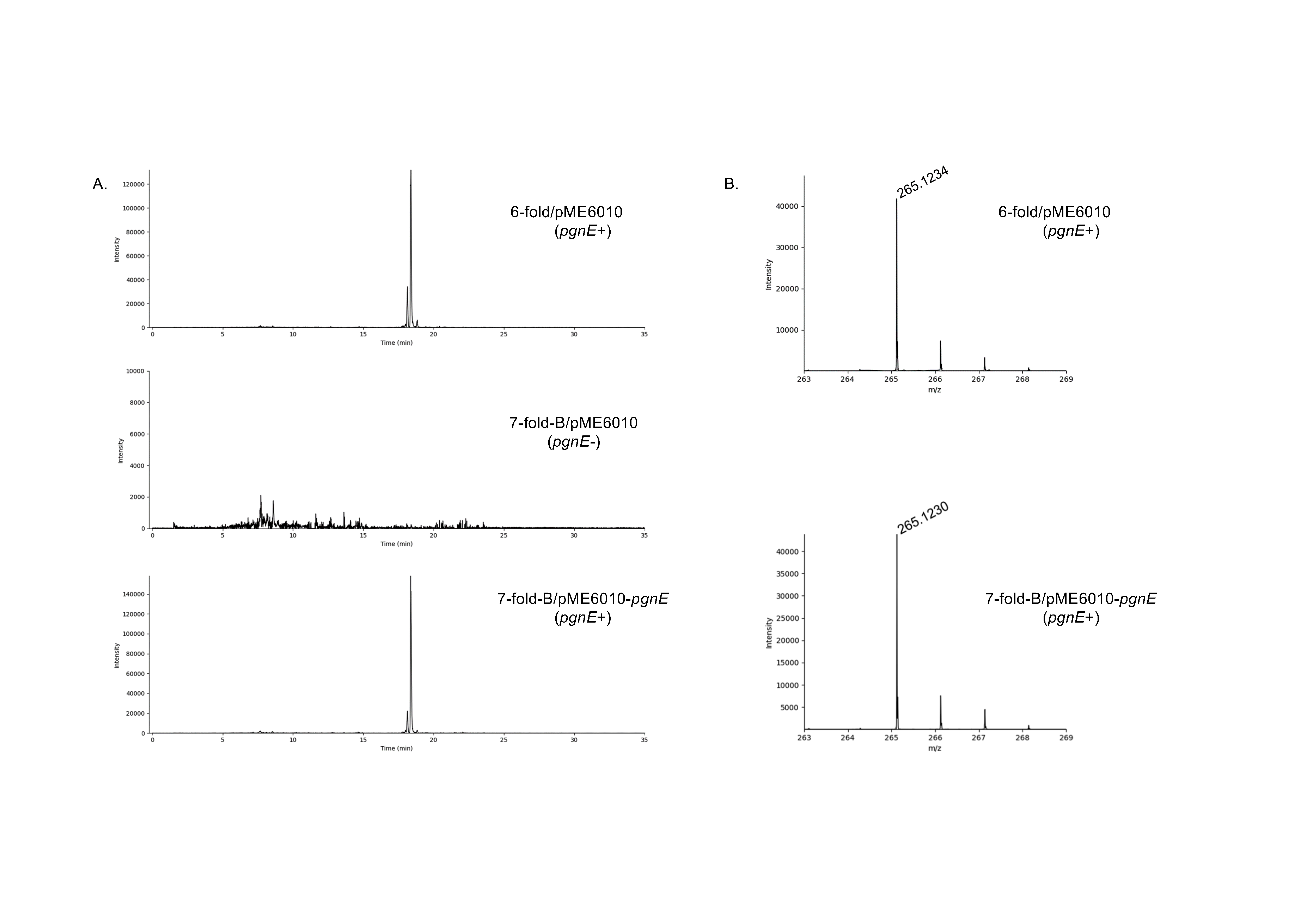


**Figure S1**. A). EIC (265.115-265.125) for protegenin A in the EtOAc extracts of 6-fold/pME6010 (top), 7-fold-B/pME6010 (middle), and 7-fold-B/pME6010-*pgnE* (bottom). B). Mass spectrum of the major protegenin A peak (obs. [M-H]^-^ = 265.1234) in 6-fold/pME6010 (obs. [M-H]^-^ = 265.1234, 0 ppm error) (top). and in 7-fold-B/pME6010-*pgnE* (obs. [M-H]^-^ = 265.1230, 1.51 ppm error) (bottom).


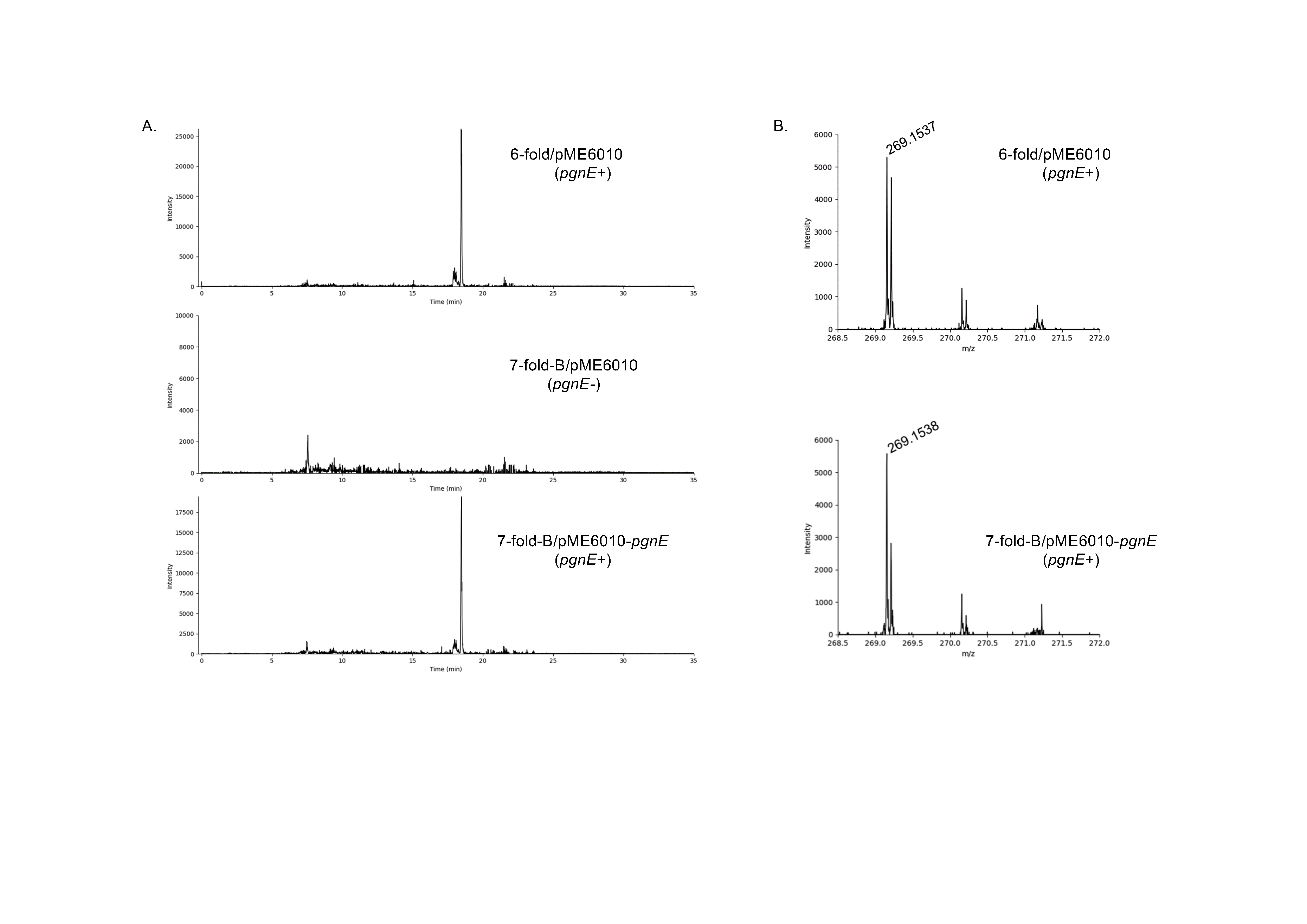


**Figure S2**. EIC (269.145-269.155) for protegenin D in the EtOAc extracts of 6-fold/pME6010 (top), 7-fold-B/pME6010 (middle), and 7-fold-B/pME6010-*pgnE* (bottom). B). Mass spectrum of the major protegenin D peak (calc. [M-H]^-^ = 269.1547) in 6-fold/pME6010 (obs. [M-H]^-^ = 269.1537, 3.72 ppm error) (top), and in 7-fold-B/pME6010-*pgnE* (obs. [M-H]^-^ = 269.1538, 3.34 ppm error) (bottom).


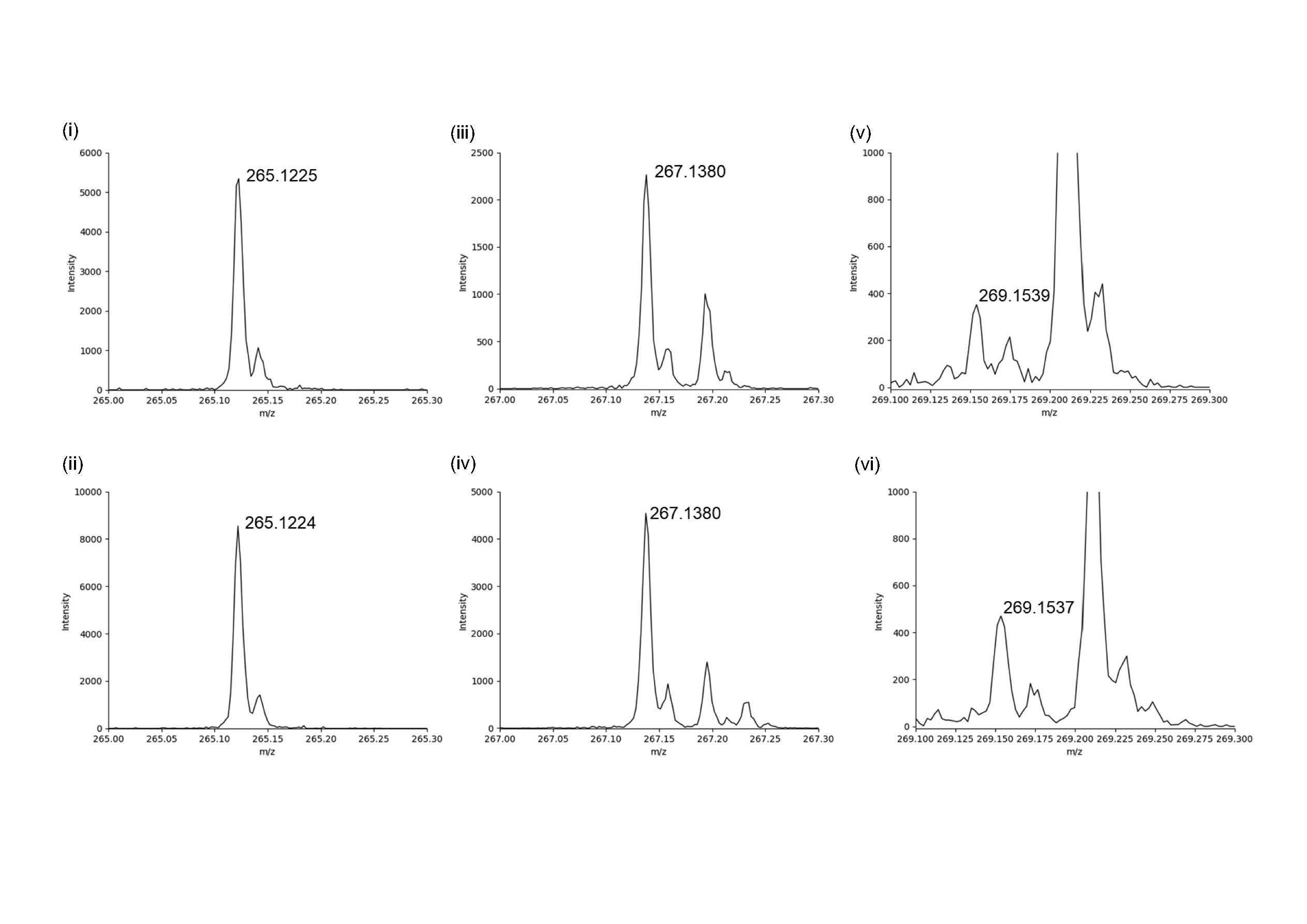


**Figure S3**. Mass spectra of protegenin A, protegenin C, and protegenin D minor peaks eluting at 18.14 min, 18.2 min, and 18.0 min respectively in the extracts of 6-fold/pME6010 and 7-fold-B/pME6010-*pgnE*. (i) Mass spectrum of protegenin A from 7-fold-B/pME6010-*pgnE* (obs. [M-H]^-^ = 265.1225, calc. [M-H]^-^ = 265.1234, 3.39 ppm error). (ii) Mass spectrum of protegenin A from 6-fold/pME6010 (obs. [M-H]^-^ = 265.1224, calc. [M-H]^-^ = 265.1234, 3.77 ppm error). (iii) Mass spectrum of protegenin C from 7-fold-B/pME6010-*pgnE* (obs. [M-H]^-^ = 267.1380, calc. [M-H]^-^ = 267.1391, 4.12 ppm error). (iv) Mass spectrum of protegenin C from 6-fold/pME6010 (obs. [M-H]^-^ = 267.1380, calc. [M-H]^-^ = 267.1391, 4.12 ppm error).(v) Mass spectrum of protegenin D from 7-fold-B/pME6010-*pgnE* (obs. [M-H]^-^ = 269.1539, calc. [M-H]^-^ = 269.1547, 2.97 ppm error). (vi) Mass spectrum of protegenin D from 7-fold-B/pME6010 (obs. [M-H]^-^ = 269.1537, calc. [M-H]^-^ = 269.1547, 3.72 ppm error).


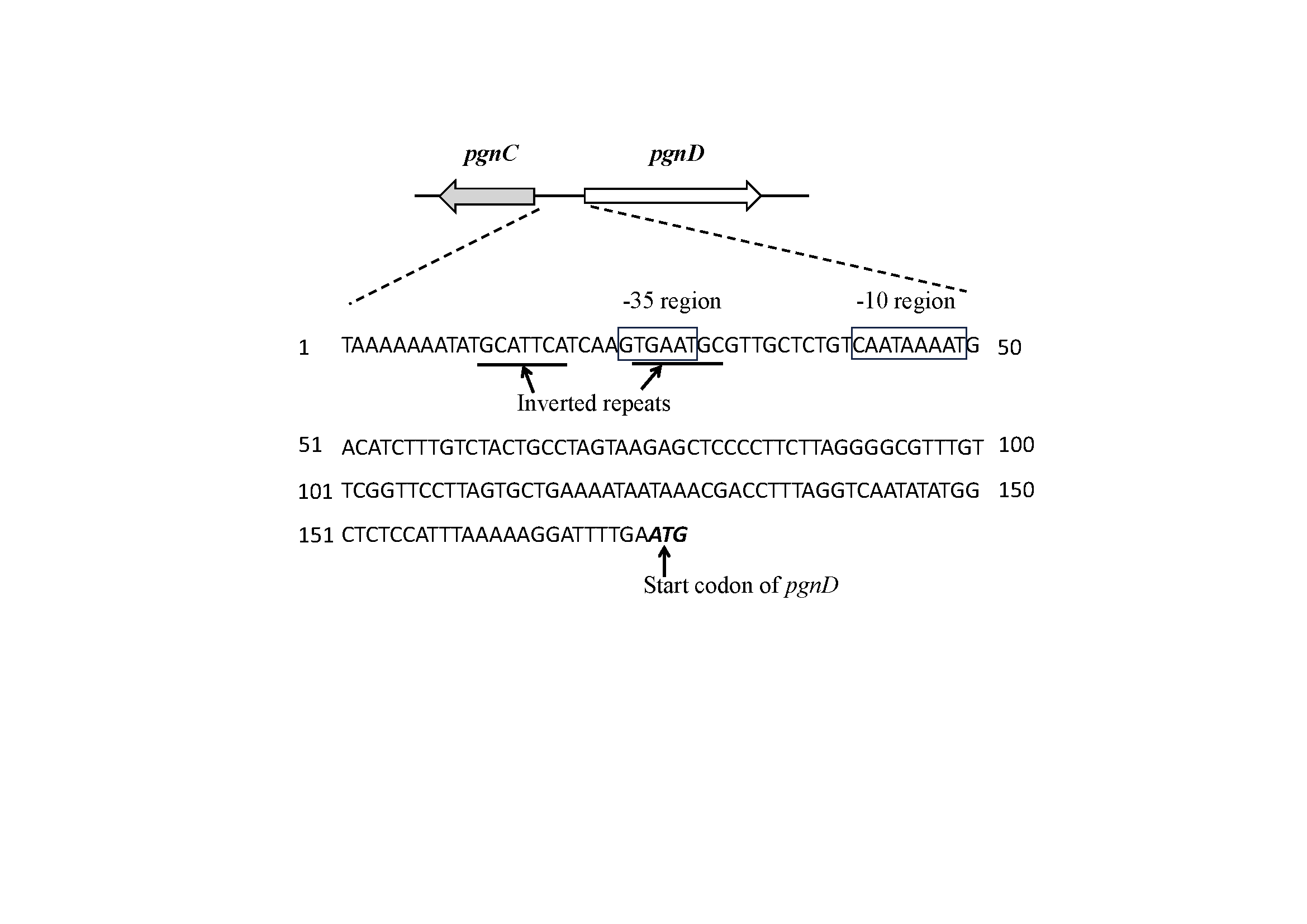


**Figure S4**. An inverted repeat (indicated by underlines) is partially overlapped with the -35 region of *pgnD* promoter. The promoter (indicated by rectangles) of *pgnD* is predicted using BPROM program (<http://www.softberry.com/berry.phtml?topic=bprom&group=programs&subgroup=gfindb>). The start codon of *pgnD* is also shown.
